# Influence of Butylated Hydroxyanisole on the Growth, Hyphal Morphology, and the Biosynthesis of Fumonisins in *Fusarium proliferatum*

**DOI:** 10.3389/fmicb.2016.01038

**Published:** 2016-06-29

**Authors:** Taotao Li, Qijie Jian, Feng Chen, Yong Wang, Liang Gong, Xuewu Duan, Bao Yang, Yueming Jiang

**Affiliations:** ^1^Key Laboratory of Plant Resource Conservation and Sustainable Utilization, Guangdong Provincial Key Laboratory of Applied Botany, South China Botanical Garden, Chinese Academy of Sciences, GuangzhouChina; ^2^University of Chinese Academy of Sciences, BeijingChina; ^3^Department of Food, Nutrition and Packaging Sciences, Clemson University, Clemson, SCUSA; ^4^Zhong Shan Entry-Exit Inspection and Quarantine Bureau, Zhong ShanChina

**Keywords:** fumonisin, *Fusarium proliferatum*, butylated hydroxyanisole, gene expression, mycelia

## Abstract

*Fusarium proliferatum* as a common fungus pathogen in foods can produce toxic fumonisins, which can cause animal diseases and increase risks of human cancers. On contrary, butylated hydroxyanisole (BHA) as a synthetic antioxidant offers a clue for preventing growth of fungal species and inhibiting production of mycotoxins. Unfortunately, information of the inhibitory mechanism of BHA on *Fusarium* species is still limited. In this study, influence of BHA treatment on growth and inhibition of fumonisin production in relation to the expression of the fumonisin biosynthesis-related genes of the *F. proliferatum* ZYF was investigated, which revealed that BHA had a negative influence on growth and fumonisin production of *F. proliferatum*. To further elucidate the mechanism of BHA on the growth of *F. proliferatum*, scanning electron microscopy (SEM) and transmission electron microscopy (TEM) were used to examine the *F. proliferatum* hyphae. The BHA treatment induced the loss of cytoplasm and cellular constituents, as well as distortion of mycelia, but it did not directly degrade the fumonisin. Furthermore, the BHA treatment markedly inhibited the expressions of *FUM1* (a polyketide synthase encoding gene) and *FUM8* (an aminotransferase encoding gene) genes, which resulted in the depression of metabolic pathway of *F. proliferatum*. The transcriptional analyses of the *FUM1* and *FUM8* genes confirmed a correlation between the fumonisin production and its gene expression. This study provided some insights into mechanisms of production of fumonisin and feasible prevention to reduce fumonisin contamination in favor of human and animal health.

## Introduction

*Fusarium proliferatum* that is commonly found in fruits such as banana (Anthony et al., 2004) and mango (Zhan et al., 2010) is a fungal pathogen, of which infection could induce a rapid deterioration of fruits (Li et al., 2012). In addition, many mycotoxins, such as fumonisins, moniliformin, beauvericin, fusaproliferin, fusaric acid, and bikaverin, can be produced by *F. proliferatum*. Li et al. (2012) reported that the *F. proliferatum* isolated from banana peel produced a large amount of fusaric acid (5-butylpicolinic acid). Besides, *F. proliferatum* isolated from banana fruit could yield a large amount of fumonisin B1 (FB1), rather than other mycotoxins present in corn and rice cultures (Jimenez et al., 1997). Due to the genetic variation, different *F. proliferatum* variants can produce different mycotoxins in food and fruits. Regardless of the significant progress on development of rapid, sensitive and robust assays to detect the presence of mycotoxins, there lacks enough efforts to reduce or even eliminate the presence of the mycotoxins in foods and feeds (Yang et al., 2014). Thus, it is worthy to investigate effective and practical means to inhibit the growth of *F. proliferatum* so as to reduce relevant mycotoxins in food.

Fumonisins can be produced by many *Fusarium* species including *F. proliferatum*, and act as one of the most harmful fungal toxins, which can cause animal diseases and human esophageal cancer (Marasas, 2001). Among these fumonisins, B-type fumonisins are the most toxic. FB1 accounts for almost 70%, while FB2 and FB3 make up about 10–20% of the total fumonisin content (Nelson et al., 1993). In recent years, some studies have reported the presence of fumonisin B1 or B2 in some fruits (Karbancioglu-Güler and Heperkan, 2008; Senyuva and Gilbert, 2008). In addition, it was found that *FUM1* (a polyketide synthase gene) encoding the β-ketoacyl synthase domain catalyzed the synthesis of the linear polyketide of fumonisins (Fanelli et al., 2012), while *FUM8* (an aminotransferase encoding gene) involved in the last step of fumonisin biosynthesis (Seo et al., 2001). Moreover, substantial information has been accumulated in regards of the key gene expression of the fumonisin biosynthesis by the *Fusarium* species (Lopez-Errasquin et al., 2007). On the other hand, although some factors to affect the fumonisin production and *FUM* gene expression of *F. proliferatum* have been reported (Samapundo et al., 2005; Kohut et al., 2009; Marín et al., 2010), little information is available on their relationship. Therefore, it is necessary for further investigation to understand and elucidate the mechanisms in controlling fumonisin biosynthesis.

Some compounds could inhibit the growth of *F. proliferatum*, but they might not be efficient in reducing production of toxins (Torres et al., 2003). For instance, a recent study reported that cinnamon oil could be a promising candidate in controlling FB1 production in corn products (Xing et al., 2014a). In addition, a synthetic antioxidant butylated hydroxyanisole (BHA) is commonly used in industrial processing for protection of cereals, grain products, cooking oils, canned goods, snacks, and many other foods (Fung et al., 1977). BHA was found to have inhibitory effects on growth, sporulation, pigmentation, and toxigenesis of *Aspergilius flavus* (Fung et al., 1977). Other researches indicated that BHA could inhibit *Fusarium verticillioides* and *F. proliferatum* populations and then reduce fumonisin production on maize grain (Torres et al., 2003; Farnochi et al., 2005). Recently, the role of BHA in controlling *Fusarium* growth and fumonisin production was well reviewed by Alberts et al. (2016). Our preliminary study also indicated that BHA could significantly inhibit the growth of *F. proliferatum* and its fusaric acid production in the culture medium. However, different species or strains could have different responses to environment factors (Jimenez et al., 2003). Nevertheless, these studies have not established a link between the fumonisin production and the expression level of fumonisin biosynthetic genes. To the best of our knowledge, the effects of BHA on the expression of the fumonisin biosynthesis-related genes and anti-fungal mechanism of *F. proliferatum* have not been investigated. Therefore, the major objective of this study was to investigate the influence of BHA on the growth, morphology and fumonisin (FB1 and FB2) production, as well as *FUM* gene expression by *F. proliferatum.* Besides, this study is expected to take early preventive actions to reduce the fumonisin contamination in an effort to protect human and animal health. On the other hand, better understanding of the inhibitive mechanism of BHA against pathogens might provide new clue for finding new targeted action of novel fungicides.

## Materials and Methods

### Fungal Strain and Growth Condition

*Fusarium proliferatum* ZYF was isolated from carambola and then stored in 50% glycerol at -80°C. The strain was grown for 7 days at 28°C on potato dextrose agar (PDA) (Oxoid, Basingstoke, Hampshire, England) in 90-mm Petri dishes. Six small agar disks (5 mm) with mycelia were taken by punching bear from colony edge and then transferred to the Czapek’s broth (CB) medium (3.0 g/L NaNO_3_, 1.0 g/L K_2_HPO_4_, 0.5 g/L MgSO_4_⋅7H_2_O, 0.5 g/L KCl, 0.01 g/L FeSO_4_, and 30 g/L sucrose, sterilized previously at 121°C for 20 min) in the presence and absence of 0.5 mM BHA (Aladdin, Shanghai, China). In this study, a concentration of 0.5 mM of BHA was chosen based on our previous experiments since this concentration could significantly inhibit the growth of *F. proliferatum*. The conical flask containing 200 ml of the above-mentioned cultures was incubated at 28°C for 10 days in an orbital shaker at 200 rpm. Growth curves were expressed in an arbitrary unit by the method of Kohut et al. (2009). Experiments were repeated at least three times.

### Scanning Electron Microscopy and Transmission Electron Microscopy

For the scanning electron microscopy (SEM) and transmission electron microscopy (TEM) experiments, the ZYF strains were placed into the potato dextrose agar in 90-mm Petri dishes in the presence and absence of 0.5 mM BHA, and then maintained for 10 days at 28°C. The diameters of the colonies were measured in two directions at a right angle to analyze the growth and morphology of ZYF according to the method of Fanelli et al. (2012). The samples for the SEM and TEM experiments were conducted after 10 days of fungal growth.

For the SEM examination, several segments (about 5–10 mm) of the cultures grown on the PDA plates were cut and immobilized in vials containing 2.5% glutaraldehyde and 2% paraformaldehyde for at least 48 h at 4°C. These samples were then washed twice with 0.1 M phosphate buffer saline, fixed with osmic acid for 2.5 h, washed again with the 0.1 M phosphate buffer saline and then dried sequentially for 10 min in a series of ethanol (30, 50, 70, 80, 90, and 100%, v/v). Following the dehydration, the samples were washed with threitol for three times (10 min each) and then frozen in a lyophilizer. Finally, these samples were coated for 180 s with 20 mA gold-palladium electroplating in a sputter coater (JFC-1600, JEOL, Japan). All samples were examined by a JSM-6360 LV SEM (JEOL, Japan) at 15 kV.

The same 10-day-old fungal cultures on the PDA plates were used for the TEM observations. The dehydration step was adopted as same as that for the SEM, then the samples were successively treated with epoxypropane, epoxypropane:resin (EPON 812, SPI, USA) (3:1), epoxypropane:resin (1:1), and epoxypropane:resin (1:3) for 1 h. Finally, these samples were soaked in 100% resin overnight and then embedded into the resin for 36 h at 60°C. Ultrathin samples were made by ultramicrotome (Leica UC6, Germany) and mounted on copper grids. After being stained with 2% uranyl acetate and lead citrate for 30 min, respectively, these samples were examined carefully using a transmission electron microscope (JEM-1010) (JEOL, Japan) at an accelerating voltage of 100 kV.

### Fumonisin Extraction and LC–MS/MS Analysis

Fumonisin was extracted by the method of Seefelde and Humpf (2002). The culture filtrates (50 ml) were collected at the 7- and 10-day and then extracted in a 100 ml solution of methanol/water (3:1, v/v) (HPLC-grade, ANPEL, Shanghai, China) using a double-layer filter paper. After modifying the pH to 5.8–6.5, the filtrate was purified with a SAX (strong anion exchange) column (500 mg, 6 ml, ANPEL, Shanghai). The SAX column was activated previously with 8 ml methanol and methanol/water (3:1, v/v) successively. The filtrate (8 ml) through the column was purified with 8 ml methanol/water (3:1, v/v) and methanol successively. Finally, the column was eluted with 10 ml methanol containing 1% acetic acid. The obtained eluant was concentrated to an appropriate volume by N_2_ purge, and then methanol/water (60:40, v/v) was added to a final volume of 1 ml. The solution was filtered through a 0.22-μm Millipore membrane filter prior to the ultra performance liquid chromatography (UPLC)–MS/MS analysis.

The analyses of FB1 and FB2 were conducted by an AB-SCIEX TRIPLE QUAD^TM^ 5500 UPLC–MS/MS system (AB SCIEX, USA). The obtained sample (10 μL) was injected onto an Ekspert 100 UPLC column (C18 column, 100 × 2.1 mm, 3 μm particle size, Thermo, USA). An optimized gradient of mobile phase (A: acetonitrile and B: 5 mM ammonium acetate) was used (**Table 1**). N_2_ was used as a nebulizing air at nebulizer temperature of 450°C. Positive ionization was selected for mass spectrometric (MS) detection. A multiple reaction monitoring (MRM) function was employed for quantification, with the fragment ions at *m*/*z* 722.5 for FB1 and 706.4 for FB2, respectively. The MS conditions were optimized for both FB1 and FB2, including declustering potential (60 V), entrance potential (10 V), collision exit potential (14 V), collision energy (50 V), and ion spray voltage (5500 V). The detection limit of FB1 and FB2 was 0.1 ng ml^-1^. The additional parameters of the analyses of FB1 and FB2 are given in Supplementary Table S1.

**Table 1:** Ultra performance liquid chromatography (UPLC) gradient program and pump time.

Time (min)	Flow rate (ml/min)	Fraction A (%)	Fraction B (%)
0–0.5	0.4	10	90
0.5–8	0.4	10–50	90–50
8–8.5	0.4	50–10	50–90
8.5–9	0.4	10	90

### RNA Isolation, Reverse Transcription and Real-Time q-PCR

For RNA isolation, the mycelia of 10-day-old ZYF grown in CB were collected. The total RNA was extracted using the Hipure Fungal RNA Mini Kit (Magen, China), according to the manufacturer’s instructions, and then stored at -80°C. The first strand cDNA was synthesized using the PrimeScript^TM^ RT Master Mix (TAKARA-RR036A, Dalian, China) subjected to RT-PCR amplification. Real-time PCR assays were used to quantify the *FUM1* and *FUM8* expressions in *F. proliferatum* strain using the primer pairs described by Kohut et al. (2009), which is listed in Supplementary Table S2.

SYBR Premix Ex Taq^TM^ mix (TaKaRa, Dalian, China) was used as a reaction mixture by adding 10.0 μl of SYBR Premix Ex Taq^TM^, 0.4 μl of PCR forward primer (10 μM), 0.4 μl of PCR reverse primer (10 μM), 0.4 μl of ROX reference dyeII and 2 μl (20 ng) of cDNA in a final volume of 20 μl. The real-time PCR was performed with a 7500 Fast Real-Time PCR System (Applied Biosystems, Foster City, CA, USA). The 7500 Fast System Software 2.0.1 was used to analyze data in fluorescent intensity. Amplification conditions were kept for 3 min at 95°C, followed by 40 cycles at 95°C for 5 s, 60°C for 5 s and 72°C for 34 s. The comparative Ct (^ΔΔ^CT) method was performed to determine the relative change in the *FUM1* and *FUM8* expressions.

### Release of Cellular Materials from *F. proliferatum*

The method of Paul et al. (2011) was used to measure the release of cellular materials. After *F. proliferatum* ZYF was cultured for 10 days in the PDB and then the mycelia were collected by centrifugation at 4000 × *g* for 20 min, washed three times and re-suspended in 50 ml of 0.1 M phosphate buffer saline (pH 7.0). The suspensions in the presence and absence of 0.5 mM BHA were incubated with agitation in an environmental incubator shaker for 0, 30, 60, and 120 min at 28°C. Samples (2 ml) was collected, then centrifuged at 12,000 × *g* for 2 min and finally 1 ml of the supernatant was used to determine the concentration of the released materials.

### Effect of BHA on FB1 Degradation

In order to investigate whether BHA could degrade FB1 directly, FB1 was added to acetonitrile/water (1:1, v/v) resulting in a concentration of 1 μg/ml in the presence and absence of 0.5 mM BHA, according to the method of Xing et al. (2014a). The sample was shaken for 48 h at 200 rpm and 25°C, extracted and detected by UPLC–MS/MS described above. The degradation experiment was also preceded in the CB medium. FB1 was added into 100 ml of CB in the presence and absence of 0.5 mM BHA and then incubated at 28°C for 48 h with shaking at 200 rpm. FB1 was extracted and detected as the above-mentioned method.

### Statistical Analysis

The SPSS 16.0 software (SPSS Inc., Chicago, IL, USA) was used for the statistical data analysis. The significant differences of growth rate and release of cellular materials from *F. proliferatum* among different culture time were analyzed by one-way analysis of variance (ANOVA). One-sample *t*-tests were used to compare the fumonisin production and *FUM* gene expression between the control and BHA-treated samples. The relationships between *FUM* gene expression and fumonisin production were evaluated using the Pearson correlation coefficients. The statistical significance was set at *p* < 0.05.

## Results

### Growth Assessment

**FIGURE 1:**
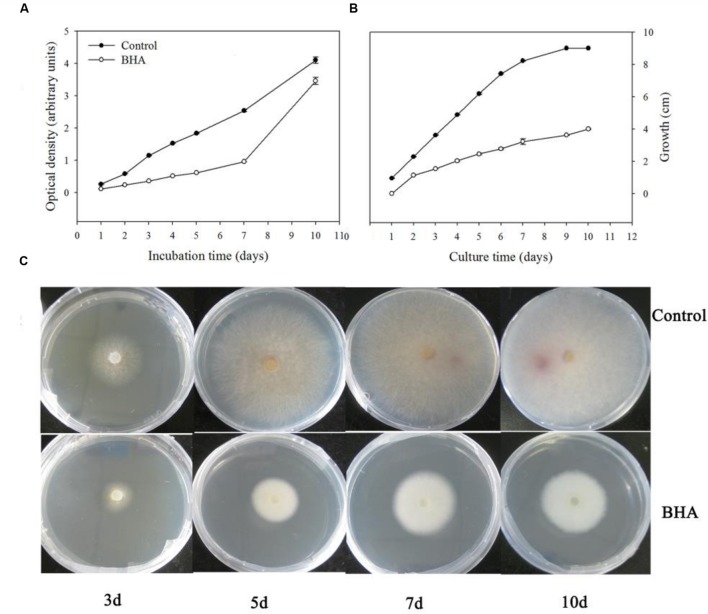
**Effect of BHA treatment on growth rates of *Fusarium proliferatum* ZYF incubation in Czapek’s broth (CB) medium (A) and PDA plate (B,C).** Values of optical densities were expressed in arbitrary units while diameters of the colonies were expressed as cm. Vertical bars indicated standard errors (*n* = 3). Experiments were repeated three times with the same results.

It was observed that BHA significantly (*p* < 0.01) inhibited the growth of *F. proliferatum* ZYF both in the CB medium (**Figure 1A**) and PDA plate (**Figure 1B**). The inhibitory effect took place during the whole culturing period although a lag phase appeared at the late stage when *F. proliferatum* was grown on the PDA without 0.5 mM BHA, due to the petri dishes restriction (90 mm) (**Figures 1B,C**). Interestingly, the growth rate of *F. proliferatum* in the CB medium increased at the late stage in the BHA-treated sample, but the growth of *F. proliferatum* was still inhibited by the BHA treatment compared with that of the control. In comparison with the growth, slight changes in the morphology of the colonies were observed (**Figure 1C**).

### Scanning Electron Microscopy and Transmission Electron Microscopy

**FIGURE 2:**
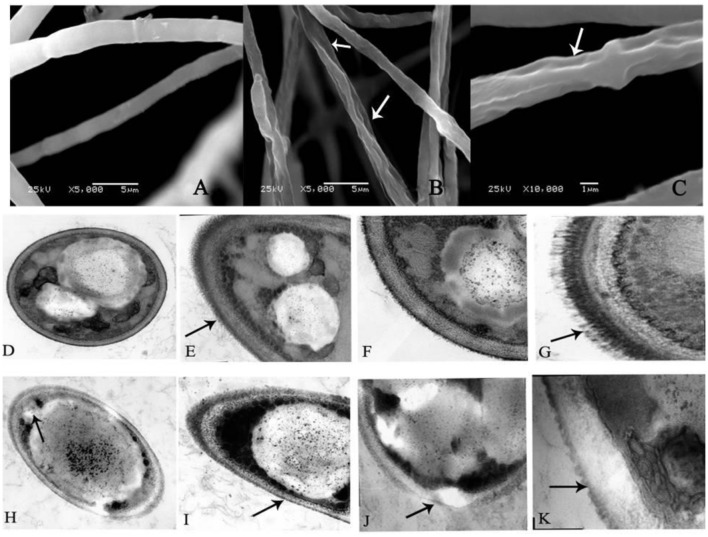
**Scanning electron microscopy (SEM) **(A–C)** and transmission electron microscopy (TEM) **(D–K)** images of *F. proliferatum* ZYF. (A)** Mycelia of non-butylated hydroxyanisole (non-BHA)-treated (control) *F. proliferatum* ZYF after 10 days of culture; **(B,C)** The BHA-treated *F. proliferatum* ZYF after 10 days of culture. **(D–G)** Mycelia of non-BHA-treated (control) *F. proliferatum* ZYF after 10 days of culture (20, 30, 50, and 150×, respectively). **(H–K)** Mycelia of the BHA-treated *F. proliferatum* ZYF after 10 days of culture (20, 30, 50, and 150×, respectively). Arrows referred to the morphologic changes in the hyphae.

As shown in **Figure 2A**, the fungus grown on the PDA plate in the absence of 0.5 mM BHA showed normal, tubular, regular and homogrnous hyphae. On the contrary, the mycelia of *F. proliferatum* ZYF treated with 0.5 mM BHA showed significant changes in the hyphal morphology. The mycelium was shown in swelled and slender appearance while the swelled mycelium was malformed, indicating the collapsed cells were caused by lack of cytoplasm (**Figure 2B**), and had depressed hyphal surfaces (**Figure 2C**). Craters were also observed on the cell walls.

To further explain the inhibitory mechanism caused by the BHA treatment, the ultrastructure of the *F. proliferatum* ZYF was investigated by TEM. The cells of *F. proliferatum* ZYF in the absence of 0.5 mM BHA showed intact cell structure (**Figure 2D**) and thick cell wall (**Figures 2E,F**), while the filiform was regular and thick (**Figure 2G**). On the contrary, the BHA treatment clearly induced turbulence of cellular structure and plenty of transparent inclusion with wide vacuoles in the cell center and large lipid globules beside the cellular walls (**Figures 2H–K**). Furthermore, the treatment remarkably resulted in disruption (**Figure 2J**) and alteration in cell walls (**Figure 2I**). Meanwhile, cell membrane damage of *F. proliferatum* ZYF was also observed (**Figure 2H**), resulting in obvious loss of cytoplasm and release of cellular materials. Additionally, the filiform became irregular and thin (**Figure 2K**).

### Effect of BHA on Fumonisin Production and FB1 Degradation

**FIGURE 3:**
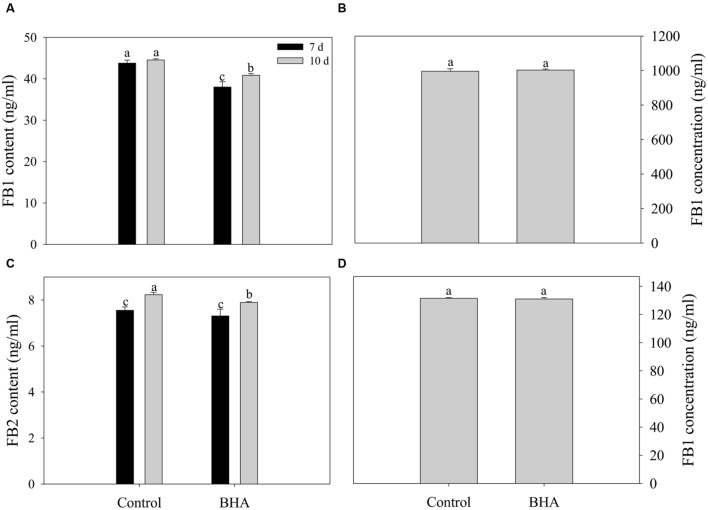
**Effects of BHA on extracellular production of FB1 **(A)** and FB2 **(B)** of *F. proliferatum* ZYF and the FB1 degradation when FB1 was incubated in acetonitrile/water **(C)** and CB media (D).** Vertical bars indicated standard errors of three independent experiments.

Both FB1 and FB2 were obtained from the CB culture in the presence and the absence of 0.5 mM BHA. The results of the UPLC–MS/MS quantification are presented in **Figures 3A,B** while the MS spectra for FB 1 and FB 2 are given in Supplementary Figure S1. After 10 days of culturing, the amount of FB1 was 44.53 ± 0.29 ng/ml for the control and 40.83 ± 0.40 ng/ml for the BHA-treated sample. For FB2, the contents in the control and BHA-treated samples were 8.13 ± 0.10 and 7.89 ± 0.04 ng/ml, respectively. Our results indicated clearly that the contents of FB1 and FB2 of the BHA-treated sample of *F. proliferatum* ZYF were significantly (*p* < 0.05) lower than those of the control sample after 10 days of culture. In order to explain further the effect of BHA on the synthesis of fumonisin, the production levels of FB1 and FB2 after 7 days of culture in the CB medium were determined. As shown in **Figures 3A,B**, BHA treatment significantly (*p* < 0.05) reduced the content of FB1 while had no significant effect on FB2 content after 7 days of culture. Additionally, BHA treatment had no direct effect on the FB1 degradation in this study (**Figures 3C,D**). After 48 h of incubation in acetonitrile/water (1:1, v/v) solution, no significant difference was observed in regards of the FB1 concentration, with 996.00 ± 14.69 ng/ml and 1003.00 ± 5.72 ng/ml for the control and BHA-treated samples, respectively, while the content of FB1 was 131.50 ± 0.50 ng/ml and 131.00 ± 1.00 ng/ml for the control the BHA-treated samples in the CB medium, respectively.

### Expressions of *FUM1* and *FUM8*

**FIGURE 4:**
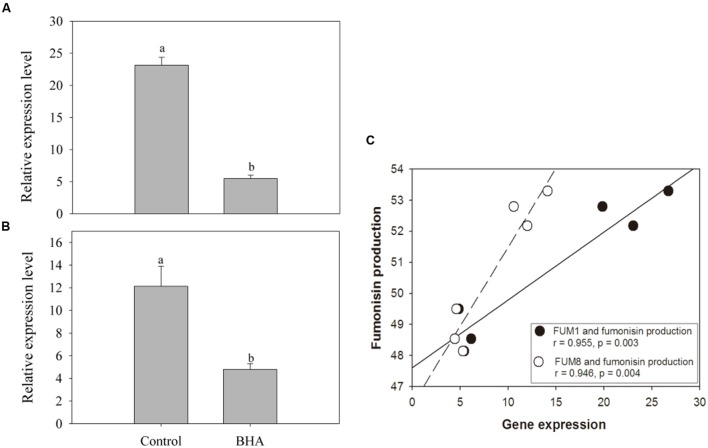
**The expression levels of *FUM1***(A)** and *FUM8***(B)** of *F. proliferatum* ZYF, and a correlation between fumonisin production and *FUM* gene expression (C).** Induction of *FUM* genes was monitored by real-time PCR. Data calculated by using the ^ΔΔ^CT method were expressed as relative units. The data were means of three independent repetitions. Vertical bars indicated standard errors.

The expression analyses of *FUM1* and *FUM8* are shown in **Figures 4A,B**. Compared with the genes in the control sample, the expressions of *FUM1* and *FUM8* genes were reduced significantly (*p* < 0.01) after the BHA treatment. The expression levels of *FUM1* and *FUM8* of the control sample were more than 4 and 2 folds higher than the BHA-treated sample, respectively. Furthermore, both *FUM1* and *FUM8* expressions exhibited significant (*p* < 0.01) correlations with the fumonisin contents in the control and BHA-treated samples (**Figure 4C**).

### Release of Cellular Contents

**FIGURE 5:**
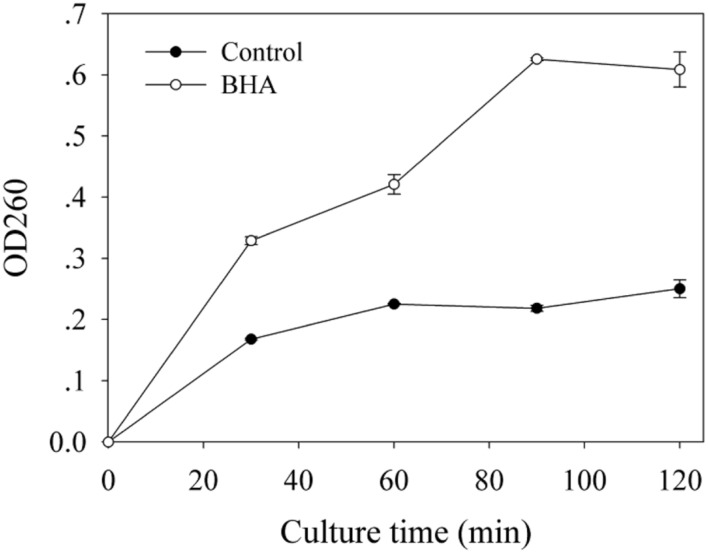
**Influence of BHA on release of cell materials.** Data were presented as means ± standard errors (*n* = 3).

Based on the absorbance value at 260 nm, the release of cellular materials from *F. proliferatum* ZYF are presented in **Figure 5**. BHA treatment increased significantly (*p* < 0.01) the release of cellular materials during the whole culture period, which could account for the growth inhibition of *F. proliferatum* ZYF.

## Discussion

*Fusarium proliferatum* can cause the diseases of some economically important fruits after harvest. Moreover, fumonisin production by the *F. proliferatum* can have a serious effect on human and animal health. With an increasing concern on food safety, many researchers focus on understanding of the molecular mechanism involved in fumonisin production in order to develop efficient strategic solutions to reduce the fumonisin contamination in fruits. Velluti et al. (2003) reported that cinnamon oil had an inhibitory effect on the *F. proliferatum* growth. On other hand, light, nutritional and environmental factors were reported to have different effects on the growth and fumonisin production of *F. proliferatum* (Kohut et al., 2009; Marín et al., 2010; Fanelli et al., 2012). The present study indicated that the BHA treatment could also inhibit the growth and reduced the fumonisin production and the gene expression of *F. proliferatum* ZYF, and the role of BHA treatment in inhibiting FB1 production can be indirect.

Butylated hydroxyanisole was proven to be able to effectively inhibit the mycelium growth too (Torres et al., 2003; Farnochi et al., 2005). Fung et al. (1977) hypothesized that BHA as a phenolic compound might interact with cell membrane proteins to cause disruption of the membrane structure. Recently, Tao et al. (2014) indicated that the inhibition of the mycelium growth of *Penicillium italicum* after a citral treatment was due to the membrane damage. In the present study, BHA treatment can effectively inhibit the mycelium growth of *F. proliferatum* both in solid and liquid media (**Figure 1**). However, there was a lag phase when *F. proliferatum* was cultured on the PDA plate in the absence of 0.5 mM BHA. In fact, after 7 days of culture, *F. proliferatum* in the absence of BHA grew to reach the edge of the Petri dishes (**Figures 1B,C**). To further monitor the growth of *F. proliferatum* on the PDA plate in the presence of BHA, the experiment was extended to 10 days. Thus, due to the restriction of the Petri dishes, slowed growth of *F. proliferatum* in the absence of BHA was observed while the BHA-treated *F. proliferatum* appeared to increase at the late culture stage (7–10 days). Similar phenomenon was also reported in *Aspergillus* spp. and other *Fusarium* species (Farnochi et al., 2005). Moreover, the growth of some fungi can be influenced by the interaction factors, such as antioxidant dose, water potential and temperature (Passone et al., 2012). Additionally, the growth of *F. proliferatum* could be influenced by different environmental factors, such as oxygen and nutrition. In the present study, it could be suggested that slow growth of *F. proliferatum* by the BHA treatment at the early stage might reduce the oxygen and nutrition consumption which might contribute to the cell growth of the BHA-treated *F. proliferatum* at the later stage. However, these suggestions need further confirmation in the future study.

The SEM observation further confirmed the significant change in morphological structure of *F. proliferatum* in the presence of BHA. The mycelia appeared swelled and collapsed (**Figures 2B,C**). This result was in agreement with the report that *F. verticillioides* exhibited the disruption of mycelia in the presence of essential oils (Xing et al., 2014b). Furthermore, *F. proliferatum* in the absence of BHA showed intact mycelia with normal structure, endoplasmic reticulum, and vacuoles within the cytoplasm, but the BHA-treated mycelia exhibited significant alterations in the hyphae including plasma membrane disruption (**Figure 2H**), thin cell walls (**Figure 2I**) or loss of integrity and rigidity of cell walls (**Figure 2J**). Irregular filiform of the BHA-treated *F. proliferatum* was also observed (**Figure 2K**). Previous study reported that the ultrastructure of fungi could be modified after the essential oil and citral treatments (Khan and Ahmad, 2011; Tao et al., 2014; Xing et al., 2014b), such as damages to cell walls, membranes and cytoplasmic contents. Additionally, loss of cellular materials confirmed further the membrane damage of *F. proliferatum* by the BHA treatment (**Figure 5**). Bajpai et al. (2013) reported that loss of cellular components could result from the irreversible damage of the cytoplasmic and plasma membranes.

*FUM1* as the first gene identified in the fumonisin biosynthesis encodes a polyketide synthase which can catalyze the synthesis of the linear polyketide of fumonisins (Fanelli et al., 2012). *FUM1* expression can be induced and repressed by NaCl at low and high concentrations (Jurado et al., 2008). For *F. proliferatum*, *FUM1* transcription was induced under non-ionic water stress at 15–35°C in *F. proliferatum* culture (Lazzaro et al., 2012a). *FUM8* as an aminotransferase gene is transcriptionally regulated also (Seo et al., 2001). Although Susca et al. (2010) reported that the presence/absence of *FUM8* was not correlated with FB2 production. However, FB1 production of *F. proliferatum* was associated with the expression level of *FUM8* under the N-stress condition (Kohut et al., 2009). In this study, fumonisin concentrations measured during culture of *F. proliferatum* ZYF paralleled with the expression levels of *FUM1* and *FUM8* (**Figures 3A,B, and 4A,B**). Moreover, a relationship existed between expression levels for two genes and fumonisin concentrations. The BHA treatment reduced significantly (*p* < 0.01) the relative expressions of *FUM1* and *FUM8* (**Figures 4A,B**). It is also worthy of mention that, although the growth of the BHA-treated *F. proliferatum* recovered between 7 and 10 days with a continuous increase of the fumonisin production, the content of fumonisin in the BHA-treated cells were still lower than that in control sample (**Figures 3A,B**). These results indicated the defect in the synthesis of fumonisins in the presence of BHA. To further clarify the influence of BHA on the FB1 biosynthesis, the FB1 degradation experiment was performed. It was found that the BHA treatment had no direct effect on the degradation of FB1 (**Figures 3C,D**), which suggested that the influence of BHA appeared to be only at the transcriptional period. The results were in agreement with the previous reports of Lazzaro et al. (2012b) and Lozano-Ojalvo et al. (2013), who reported that the biosynthesis of mycotoxin was regulated at a genetic level. Therefore, our results suggested that reduced expression of the *FUM1* and *FUM8* genes might be involved in the decrease in fumonisin content by BHA treatment. However, it is worthy to note that *FUM1* and *FUM8* were reduced by 70–80% while only 8% and 3% decrease in FB1 and FB2 contents was observed after the BHA treatment. Similarly, previous research also indicated that the inhibition rates of aflatoxins production and *aflD* gene expression were rather different in *Aspergillus flavus* RCP08108 (Passone et al., 2012). In this study, we cautiously postulated that inhibition of *FUM1* and *FUM8* expression might contribute partially to the decrease of FB1 and FB2 contents. Besides the possible functional importance of *FUM1* and *FUM8* genes in the inhibition of fumonisin production by BHA treatment, other genes might be also regulated by the treatment and involved in the fumonisin backbone modification and ABC transport during the biosynthesis of fumonisin (Proctor et al., 2003). In this context, it is necessary for further studies using RNA-Seq-based technique to find more genes which might be involved in the genetic regulations by the BHA treatment. For example, the analysis of the expression of *FUM19*, which encodes an ABC transporter and was previously correlated to fumonisin production, would enhance to verify the role of BHA in the biosynthesis and transport of fumonisins after the BHA treatment. Moreover, further work by applying bio-omics methods, such as proteomics and transcriptomics, might be able to explain more the mechanism of the inhibitory effect of BHA on the fumonisin production.

## Author Contributions

TL and YJ conceived and designed the study. TL, QJ, YW, LG, XD, and BY performed the experiments and analyzed the data. TL, YJ, and FC drafted and revised the manuscript. All authors participated in the interpretation of data of the manuscript. All authors approved the submission and publication for all aspects of the work.

## Conflict of Interest Statement

The authors declare that the research was conducted in the absence of any commercial or financial relationships that could be construed as a potential conflict of interest.
