# Supplementary material for: Influence of Butylated Hydroxyanisole on the Growth, Hyphal Morphology, and the Biosynthesis of Fumonisins in Fusarium proliferatum
Source: Front Microbiol. 2016 Jun 29;7:1038. doi: 10.3389/fmicb.2016.01038 (PMC4942755; doi:10.3389/fmicb.2016.01038)
Supplement: Supplementary file 1 [file Image_1.PDF]

Supplemental figure legends:

Figure S1: The full MS spectra for FB 1 and FB 2. (A): MS spectra for standard FB1 and FB2; (B): MS spectra for sample choosing one sample as example.

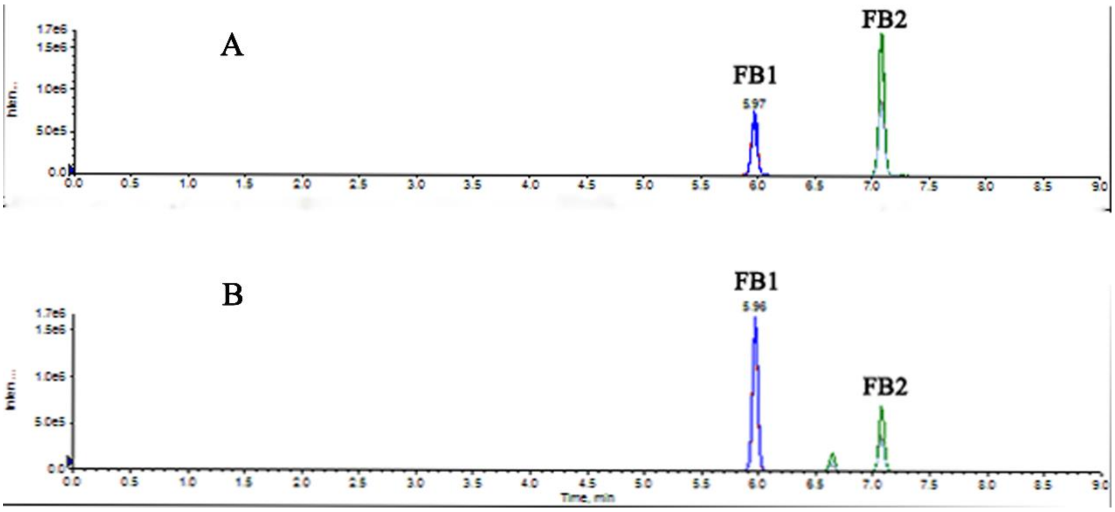

Figure S1
