# Supplementary material for: Influence of Butylated Hydroxyanisole on the Growth, Hyphal Morphology, and the Biosynthesis of Fumonisins in Fusarium proliferatum
Source: Front Microbiol. 2016 Jun 29;7:1038. doi: 10.3389/fmicb.2016.01038 (PMC4942755; doi:10.3389/fmicb.2016.01038)
Supplement: Supplementary file 2 [file Table_1.DOCX]

**Table S1** The detailed parameters of the detection of FB1 and FB2.

| Compound | Parent ion  (m/z) | Daughter ion  (m/z) | Cone voltage (eV) | Collision energy  (eV) |
| --- | --- | --- | --- | --- |
| FB1 | 722.5 | 352.4*  334.4 | 60  60 | 50  52 |
| FB2 | 706.4 | 336.4*  318.4 | 65  65 | 50  50 |

*quantitative ion
