# Supplementary material for: Influence of Butylated Hydroxyanisole on the Growth, Hyphal Morphology, and the Biosynthesis of Fumonisins in Fusarium proliferatum
Source: Front Microbiol. 2016 Jun 29;7:1038. doi: 10.3389/fmicb.2016.01038 (PMC4942755; doi:10.3389/fmicb.2016.01038)
Supplement: Supplementary file 3 [file Table_2.DOCX]

**Table S2** Primers for *FUM1*, *FUM8* and *Histone H3* gene of *Fusarium proliferatum*.

| Gene | Primer name | Sequence of primer (5’ to 3’) |
| --- | --- | --- |
| *FUM1* | Fum1-for | ACTTTGCCATTTCCAACCGTAT |
|  | Fum1-rev | GGGAGTTTTTCCATCCGAATTT |
| *FUM8* | Fum8-for | ATTCCATGAGGAGGCAATGCAG |
|  | Fum8-rev | GGTGCTATTCCTTCGAGGTCAC |
| *Histone H3* | H3-1a | ACTAAGCAGACCGCCCGCAGG |
|  | H3-1b | GCGGGCGAGCTGGATGTCCTT |
